# Effects of kaempferol on weather-related pain: an open-label pilot study of subjective headache and other discomforts in pre-intervention and intervention periods in Japan

**DOI:** 10.1007/s00484-025-02985-6

**Published:** 2025-07-24

**Authors:** Yasutaka Ikeda, Moe Yamamoto, Aina Gotoh-Katoh, Shoichiro Inoue, Jun Sato

**Affiliations:** 1 Advanced Research Institute for Core Science, Otsuka Pharmaceutical Co., Ltd, 2-16-4 Konan, Minato-ku, Tokyo, 108-8242 Japan; 2 Department of Pain Medicine, Aichi Medical University, 1-1 Yazako-Karimata, Nagakute, Aichi, 480-1195 Japan

**Keywords:** Flavonoid, Hypoxia, Kaempferol, Oxygen utilization, Parasympathetic nervous system, Weather-related pain

## Abstract

Adverse or fluctuating weather conditions can negatively affect health, causing symptoms known as weather-related pain. Although pharmacological treatments, including painkillers, are commonly used, they frequently provide only symptomatic relief and may cause side effects. Therefore, interest in non-pharmacological and dietary interventions is increasing. While inner ear sensitivity to barometric pressure fluctuations—activating the sympathetic nervous system—has traditionally been considered the main mechanism, we propose that reduced oxygen utilization due to peripheral hypoxia under low barometric pressure is also a key factor. Kaempferol, a dietary flavonoid, has been shown in prior studies to enhance oxygen utilization and promote parasympathetic nervous system dominance. We hypothesized that daily kaempferol intake would alleviate weather-related discomfort by improving oxygen use and autonomic balance. In this pilot study, 458 individuals with moderate weather-related symptoms took 10 mg of kaempferol daily for 4 weeks. Participants completed questionnaires before and after the intervention. Data from 387 individuals with over 80% adherence were analyzed. Results showed significant reductions in symptom frequency (e.g., headache: Cohen’s *d* = 0.61, *p* < 0.001), duration (rank-biserial correlation = 0.64, *p* < 0.001), and severity (Cohen’s *d* = 0.57, *p* < 0.001). By the end of the intervention, over 80% of participants reported symptom improvement. These findings suggest that kaempferol is a promising non-pharmacological strategy for managing weather-related physical and mental symptoms by targeting oxygen utilization and autonomic regulation. This trial is registered with the UMIN Clinical Trials Registry (UMIN000045066).

**Supplementary Information:**

The online version contains supplementary material available at 10.1007/s00484-025-02985-6.

## Introduction

Adverse or fluctuating weather conditions can negatively impact human health, potentially lowering the quality of life and interfering with daily activities such as commuting to work or school (Sato et al. 2021). This phenomenon, commonly referred to as meteoropathy or weather-related pain, includes various physical symptoms—such as headaches and languidness—as well as mental symptoms, including depression and irritability (Hoxha and Zappacosta 2023; Sato et al. 2021). The severity and type of symptoms vary among individuals (Lee et al. 2018; Mazza et al. 2012), and approximately 30% of the global population is estimated to experience weather-related pain—a figure expected to rise with the increasing frequency of extreme weather events (Lickiewicz et al. 2020). In addition to its health burden, weather-related pain can reduce work productivity and contribute to both presenteeism and absenteeism, resulting in broader societal and economic consequences. Notably, females appear to be more susceptible to those symptoms, which may further limit their participation in daily and professional activities (Lee et al. 2018). Addressing this growing public health concern requires the development of effective and accessible interventions.

Among meteorological factors, barometric pressure fluctuations are considered the most influential in triggering symptoms (Dixon et al. 2019; McGorry et al. 1998; Okuma et al. 2015; Otsuka and Sato 2023). In Japan, a forecasting service called TENKITSU YOHOU^®^ (Weathernews Inc., Japan) predicts pain episodes based on the following three types of barometric pressure fluctuations: micro-pressure fluctuations, synoptic-scale low-pressure systems, and semidiurnal atmospheric tides (Otsuka and Sato 2023). The prevailing hypothesis suggests that the inner ear’s heightened sensitivity to these fluctuations activates the sympathetic nervous system, leading to pain and discomfort (Sato 2015). While exposure to micro-high barometric pressure (mHBP) has been shown to alleviate symptoms (Sakurai et al. 2022), access to this treatment is limited to specific facilities. Consequently, many individuals rely on over-the-counter painkillers, which offer only temporary relief and may cause adverse effects, including gastrointestinal issues or medication overuse headaches (Zwart et al. 2003). Alternative approaches—including acupressure, herbal remedies, and earplugs—have been explored; however, their efficacy remains inconsistent.

Recently, improving peripheral hypoxia has emerged as a promising therapeutic strategy. mHBP exposure not only reduces inner ear sensitivity but also enhances oxygen delivery, lowers heart rate, and promotes parasympathetic nervous system activity—especially when combined with high oxygen concentrations (Sakurai et al. 2022). These findings suggest that improving oxygen utilization efficiency is a key mechanism in managing weather-related pain. Inspired by the adaptive mechanisms of organisms living in hypoxic environments, we focused on kaempferol, a flavonoid abundantly present in various edible plants, particularly those growing at high altitudes (Mizokami et al. 2021). Flavonoids are synthesized in response to environmental stress and are known for their antioxidant and anti-inflammatory properties. Kaempferol, in particular, has demonstrated various biological activities, including anticancer, cardioprotective, and neuroprotective effects (Chen and Chen 2013; Tzeng et al. 2011; Imran et al. 2019; Wang et al. 2018; Yao et al. 2019). Our previous studies have shown that kaempferol enhances mitochondrial oxidative metabolism and increases intracellular ATP levels under hypoxic conditions by inhibiting the stabilization of hypoxia-inducible factor-1α (Mizokami et al. 2021; Akiyama et al. 2022). Among 65 phytochemicals tested, kaempferol was the most effective in this regard. Clinical trials further demonstrated that a daily intake of 10 mg of kaempferol improves oxygen utilization efficiency, reduces resting heart rate, and promotes parasympathetic nervous system dominance (Ikeda et al. 2024a, b). It has also been shown to enhance athletic performance in doses ranging from 10 to 50 mg (Okita et al. 2024).

Despite growing interest in non-pharmacological interventions, few studies have explored dietary strategies targeting oxygen utilization in the context of weather-related pain. This highlights a critical gap in the current literature and underscores the need to investigate novel, accessible approaches such as kaempferol supplementation. Based on these findings, we hypothesized that daily intake of kaempferol could serve as a practical and effective strategy for alleviating weather-related pain by improving oxygen utilization and autonomic balance, thereby reducing the physiological stress responses triggered by barometric pressure fluctuations. To test this hypothesis, we conducted an open-label pilot study involving individuals living in the southern part of the Kanto region of Japan who reported moderate weather-related headaches**—**the most common symptom of weather-related pain (Sato et al. 2021). Participants consumed 10 mg of kaempferol daily for 4 weeks. We assessed changes in subjective physical and mental symptoms using electronic questionnaires that evaluated symptom frequency, duration, and severity before and during the intervention. To account for environmental variability, participants were limited to those residing at similar latitudes, and regional weather data—including barometric pressure—were also collected. Given the absence of established biomarkers for weather-related pain, we focused on subjective symptoms that directly reflect participants’ actual experiences. To the best of our knowledge, this is the first study to evaluate kaempferol’s potential to alleviate weather-related symptoms in a real-world setting. The findings may offer valuable insights for both public health and clinical practice.

## Materials and methods

### Study design and participants

**Fig. 1:**
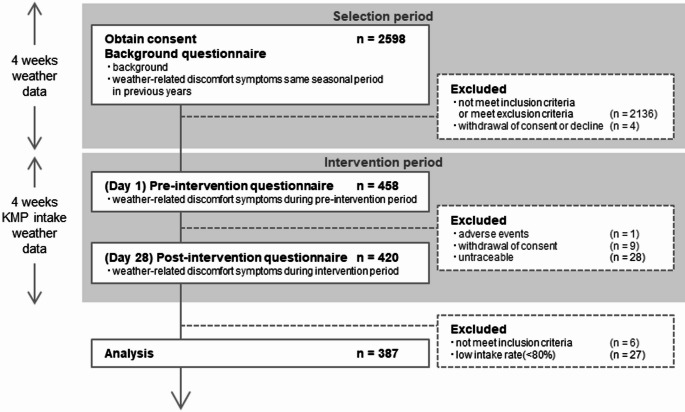
Flow diagram of the trial process.The diagram illustrates the flow of participant recruitment, eligibility screening, intervention, exclusion, and final data analysis

This study was approved by the Ethics Committee of Otsuka Pharmaceutical Co., Ltd. (approval number 2102, dated July 28, 2021) and conducted in accordance with the Declaration of Helsinki and its later amendments. It was registered with the University Hospital Medical Information Network Clinical Trials Registry (UMIN000045066) and followed the International Committee of Medical Journal Editors guidelines. This trial was conducted between August and October 2021 (Fig. 1). Participant privacy and confidentiality were strictly maintained.

Healthy adults reporting weather-related discomfort were recruited via a mobile application provided by Weathernews Inc. Informed consent was obtained from 2,598 individuals. Eligibility was determined using a background questionnaire that collected demographic data and symptom history. Participants retrospectively assessed symptoms experienced during the same seasonal period in previous years. The inclusion criteria required residence in the southern Kanto region of Japan and a history of moderate weather-related headaches. The exclusion criteria included food allergies, pregnancy, breastfeeding, plans to become pregnant, and current use of medications or treatments. Individuals not meeting inclusion criteria, meeting any exclusion criteria, or who withdrew consent were excluded.

The intervention began with 458 eligible participants who completed a pre-intervention questionnaire assessing weather-related symptoms experienced over the previous 4 weeks. This served as the baseline for subsequent comparisons. Participants then consumed one serving of the test food (10 mg kaempferol aglycone) daily after breakfast for 4 weeks and completed a post-intervention questionnaire on the final day. Intake adherence was monitored through daily self-reports and photographic documentation of empty packaging. Weekly health status reports and daily records were also collected throughout the intervention.

During the study, 38 participants discontinued due to adverse events, withdrawal of consent, or loss to follow-up. Overall, 420 participants completed the intervention, and 387 individuals with an intake adherence rate of ≥ 80% were included in the final analysis. Weather data—including barometric pressure, temperature, and humidity—were collected from 4 weeks before the intervention through its end to account for environmental variability.

**Fig. 2:**
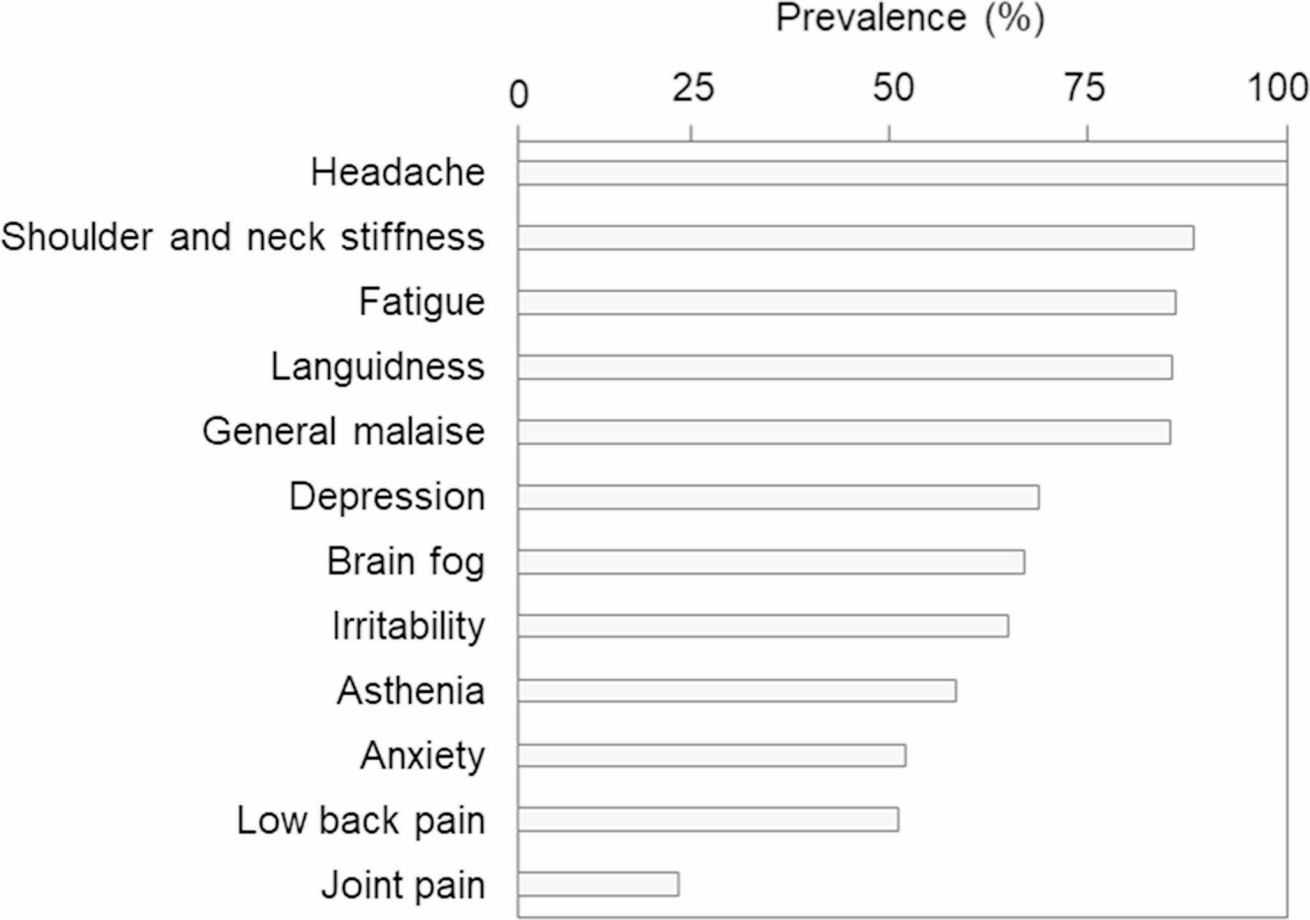
Prevalence of subjective weather-related discomfort symptoms. The reported prevalence of physical and mental symptoms—including headache, shoulder and neck stiffness, fatigue, languidness, general malaise, depression, brain fog, irritability, asthenia, anxiety, low back pain, and joint pain—based on the background questionnaire (*n* = 387)

Participant demographics are summarized in Table 1. The final analysis group was predominantly female (89%), with an average age of 38 years (range: 20–63 years). Each 10-year age group from the 20 s to 50 s was well represented. Most participants were non-smokers (91%) and reported consuming alcohol less than once a week (67%). According to the background survey, over 80% of participants experienced additional symptoms, including shoulder and neck stiffness (88%), fatigue (86%), languidness (85%), and general malaise (85%) (Fig. 2). Mental symptoms were also common, with 49% and 21% of participants reporting low back and joint pains, respectively.

**Table 1:** Participants’ characteristics

		*n*	%
Sex	Female	344	89
	Male	43	11
Age	20–29	92	24
38.3 ± 10.3	30–39	121	31
(mean ± SD)	40–49	113	29
	50–59	55	14
	60–63	6	2
Smoke	−	353	91
	+*	34	9
Alcohol	−**	261	67
	+	126	33

*< 40 cigarettes/day, ** < 1 time/week

### Selection of participants with subjective moderate weather-related headache

Participants with subjective moderate weather-related headaches were selected through a structured three-step process using a headache checklist based on a large-scale national survey on weather-related pain in Japan (Weathernews Inc. 2023).

First, participants were eligible if they selected one of the following statements regarding headache frequency:

- “I have headaches about one-third of the month,”
- “I have headaches approximately 5–10 days per month,”
- “I have headaches every day, and they worsen about one-third of the month,” or
- “I have headaches every day, and they worsen approximately 5–10 days per month.”

Second, participants were required to report weather sensitivity by selecting at least two of the following six statements:

- “I can tell it is going to rain from changes in my physical condition, such as headaches,”
- “I am sensitive to barometric pressure fluctuations,”
- “I tend to get sick at the change of seasons,”
- “I am not good with temperature differences,”
- “It is hard to get up on rainy mornings,” or
- “My mood fluctuates with the weather.”

Finally, a physician (J.S.) reviewed the checklist responses to confirm whether each participant met the criteria for moderate weather-related headaches. This multi-step process ensured consistent and reliable identification of the target population for the intervention.

### Questionnaire of subjective weather-related discomfort symptoms

Subjective symptoms associated with weather-related discomfort were assessed using an electronic questionnaire developed based on validated instruments from previous studies (Weathernews Inc., 2023; Mazza et al. 2012). The questionnaire included five major physical symptoms—headache, shoulder and neck stiffness, joint pain, lower back pain, and languidness (Weathernews Inc. 2023)—six common mental symptoms—depression, irritability, asthenia, fatigue, anxiety, and brain fog—and one physical and mental symptom: general malaise.

For each symptom, participants reported the weekly frequency, duration, and severity. Symptom duration was categorized into the following six levels: almost all day, half a day, a few hours, several tens of minutes, a few minutes, or none. Symptom severity was rated using a numerical rating scale (NRS) ranging from 0 (no symptoms) to 10 (severity sufficient to interfere with daily life).

This questionnaire was administered at three time points: during the background survey, on the first day of the intervention (pre-intervention), and on the final day of the intervention (post-intervention). The pre-intervention responses, which reflected symptoms experienced over the previous 4 weeks, served as the baseline for comparison. Post-intervention responses captured symptoms experienced during the four-week intervention period.

Changes in symptoms were categorized as “improved,” “no change,” or “worsened” based on comparisons between pre- and post-intervention responses. For frequency, “improved” and “worsened” were defined as a change of at least one occurrence per week, either an increase or decrease. For severity, they were defined as an increase or decrease of at least one point on the NRS score. Additionally, on the final day, participants were asked to evaluate their overall symptom change by comparing their experiences during the intervention period with those during the pre-intervention period, using a six-point scale: greatly improved, improved, slightly improved, slightly worsened, worsened, or greatly worsened. Each symptom-related question required a single response.

### Test food

The test food was provided in granular form and contained 10 mg of kaempferol aglycone per serving. The kaempferol aglycone was obtained by enzymatically hydrolyzing kaempferol glycosides extracted from horseradish leaf powder. To ensure stability and preserve bioactivity, the product was stored under light-shielding conditions at controlled temperatures ranging from 1 °C to 30 °C.

### Weather data

Hourly meteorological data—including barometric pressure, temperature, and humidity—were obtained from 29 observation areas located in the southern Kanto region of Japan. Data collection covered the period from 4 weeks before the intervention through its conclusion (August 29, 2021, to October 23, 2021) and was provided by Weathernews Inc. (Japan). Daily averages and fluctuations were calculated for each parameter, with fluctuations defined as the difference between the maximum and minimum values recorded within 24 h.

### Statistical analysis

To evaluate changes in symptom frequency and severity before and after the intervention, we conducted a paired *t*-test and calculated Cohen’s *d* with a 95% confidence interval (CI) to estimate effect sizes. For symptom duration, the Wilcoxon signed-rank test was used, and effect sizes were expressed as rank-biserial correlation with 95% CI.

In the Wilcoxon signed-rank test, symptom duration categories were converted to ordinal numerical values as follows: none = 0, a few minutes = 1, several tens of minutes = 2, a few hours = 3, half a day = 4, and almost all day = 5. Additionally, McNemar’s test was applied to assess categorical changes in symptom duration using two binary comparisons: (1) “almost all day” or “half a day” versus all other responses, and (2) “none” versus all other responses. Odds ratios with 95% CI were calculated to estimate the magnitude of change for each comparison.

To account for multiple comparisons across symptoms, Bonferroni correction was applied. For weather data analysis, unpaired *t*-tests were used to compare environmental conditions across periods. All statistical analyses were performed using SAS software version 9.4 (SAS Institute Inc., Cary, NC, USA), R version 4.3.3 with the “effectsize” and “rank_biserial” packages, and Python version 3.10. All hypothesis tests were two-sided, and statistical significance was defined as *p* < 0.05. Data are presented as mean ± standard deviation (SD) or standard error (SE), as appropriate.

## Results

### Weather data

Weather data comparing the pre-intervention and intervention periods are summarized in Table 2. Daily barometric pressure fluctuations were significantly greater during the intervention period than in the pre-intervention period across nearly all observation areas, with the exception of Okutama and Hakone towns (*p* < 0.05). No significant differences were observed in daily fluctuations of temperature or humidity between the two periods. However, daily average barometric pressure was significantly higher during the intervention period in all areas except Okutama town (*p* < 0.05). Additionally, the daily average temperature was significantly lower in all areas except Machida city, and the daily average humidity was significantly lower in Choshi city and Narita city during the intervention period (*p* < 0.05).

**Table 2:** Weather data for four-week pre-intervention period and four-week intervention period

	Daily barometric pressure fluctuation (hPa)		Daily temperature fluctuation (ºC)		Daily humidity fluctuation (%)		Daily average barometric pressure (hPa)		Daily average temperature (ºC)		Daily average humidity (%)	
Observation area	Pre-intervention	Intervention	Pre-intervention	Intervention	Pre-intervention	Intervention	Pre-intervention	Intervention	Pre-intervention	Intervention	Pre-intervention	Intervention
Setagaya ward	4.1 ± 1.6	6.6 ± 3.8*	5.8 ± 2.3	6.8 ± 2.5	30.7 ± 16.9	35.3 ± 13.5	1010.8 ± 3.3	1014.1 ± 5.9*	23.5 ± 2.8	20.1 ± 3.8*	78.5 ± 10.0	75.1 ± 10.4
Chuo ward	4.1 ± 1.6	6.6 ± 3.9*	5.7 ± 2.3	6.8 ± 2.5	26.0 ± 12.9	30.5 ± 12.7	1013.1 ± 3.3	1016.3 ± 5.9*	23.3 ± 2.7	19.9 ± 3.7*	80.8 ± 9.4	76.4 ± 9.9
Chiyoda ward	4.1 ± 1.6	6.7 ± 3.9*	5.9 ± 2.4	7.1 ± 2.7	28.8 ± 14.3	33.4 ± 13.5	1012.7 ± 3.3	1016.0 ± 5.9*	23.1 ± 2.7	19.7 ± 3.7*	81.2 ± 9.8	76.9 ± 10.0
Okutama town	6.7 ± 1.8	8.0 ± 3.6	7.3 ± 3.4	8.7 ± 3.9	26.1 ± 13.8	31.1 ± 16.1	927.5 ± 2.8	929.4 ± 5.5	19.4 ± 2.4	15.7 ± 3.6*	87.5 ± 9.3	83.4 ± 10.6
Hachioji city	4.8 ± 1.9	6.8 ± 4.0*	6.5 ± 3.3	8.3 ± 3.4	28.6 ± 14.2	33.5 ± 15.7	994.3 ± 3.2	997.3 ± 5.8*	22.2 ± 2.6	18.5 ± 3.7*	80.5 ± 11.3	77.9 ± 9.8
Machida city	4.2 ± 1.6	6.5 ± 3.8*	6.0 ± 2.5	7.1 ± 2.8	31.1 ± 16.2	34.6 ± 14.8	1005.5 ± 3.3	1008.7 ± 5.9*	23.0 ± 2.7	19.3 ± 3.6	78.5 ± 11.4	75.5 ± 10.4
Hakone town	9.7 ± 2.0	10.9 ± 4.0	6.7 ± 3.2	8.2 ± 3.2	25.9 ± 11.4	28.9 ± 11.2	972.9 ± 3.0	975.6 ± 5.8*	21.8 ± 2.4	18.0 ± 3.5*	81.6 ± 9.0	78.9 ± 7.9
Atsugi city	5.0 ± 2.0	7.0 ± 4.1*	6.2 ± 2.7	7.4 ± 3.1	28.0 ± 14.1	31.5 ± 12.9	1003.8 ± 3.2	1006.9 ± 5.9*	23.2 ± 2.6	19.4 ± 3.6*	80.9 ± 10.9	77.5 ± 9.4
Odawara city	5.6 ± 2.0	7.4 ± 4.2*	6.8 ± 3.1	8.1 ± 3.2	27.5 ± 11.5	29.4 ± 11.6	996.6 ± 3.1	999.7 ± 5.8*	22.9 ± 2.3	19.2 ± 3.4*	79.3 ± 8.9	76.7 ± 7.3
Chuo ward, Sagamihara city	4.5 ± 1.7	6.6 ± 3.9*	6.3 ± 3.0	7.8 ± 3.3	29.6 ± 15.2	32.8 ± 14.9	1000.4 ± 3.1	1003.5 ± 5.8*	22.4 ± 2.6	18.6 ± 3.6*	79.3 ± 11.7	76.3 ± 10.4
Chigasaki city	4.1 ± 1.6	6.6 ± 3.8*	5.8 ± 2.5	7.1 ± 2.8	25.9 ± 11.8	28.9 ± 11.9	1011.9 ± 3.3	1015.1 ± 6.0*	23.5 ± 2.5	19.9 ± 3.6*	80.2 ± 9.1	77.4 ± 8.4
Miura city	13.8 ± 2.2	14.8 ± 4.4*	5.6 ± 2.0	6.1 ± 1.7	19.3 ± 9.0	22.5 ± 9.6	1009.0 ± 3.2	1012.1 ± 6.1*	23.4 ± 2.3	20.0 ± 3.4*	80.8 ± 7.6	77.1 ± 7.1
Yokosuka city	4.0 ± 1.6	6.4 ± 4.0*	6.1 ± 2.5	6.6 ± 2.3	22.0 ± 10.1	26.4 ± 11.5	1010.3 ± 3.2	1013.4 ± 6.1*	23.2 ± 2.4	19.8 ± 3.6*	80.8 ± 8.5	76.2 ± 9.3
Nishi ward, Yokohama city	4.1 ± 1.6	6.5 ± 3.9*	5.9 ± 2.4	7.0 ± 2.7	26.5 ± 12.8	30.9 ± 11.2	1010.6 ± 3.2	1013.8 ± 6.0*	23.4 ± 2.7	19.8 ± 3.7*	79.6 ± 9.7	75.2 ± 9.8
Kumagaya city	4.3 ± 1.6	6.7 ± 3.7*	6.6 ± 3.2	7.9 ± 3.7	31.7 ± 15.1	35.0 ± 16.6	1010.3 ± 3.3	1013.6 ± 5.8*	23.0 ± 2.7	19.3 ± 3.7*	78.9 ± 10.3	75.4 ± 10.2
Urawa ward	4.2 ± 1.6	6.7 ± 3.7*	6.5 ± 3.1	7.6 ± 3.0	31.9 ± 17.9	38.4 ± 14.8	1013.0 ± 3.3	1016.3 ± 5.9*	23.3 ± 2.8	19.6 ± 3.8*	76.9 ± 10.1	74.7 ± 10.4
Chichibu city	3.9 ± 1.3	6.2 ± 3.5*	7.4 ± 3.9	8.9 ± 4.4	28.9 ± 14.6	35.7 ± 16.4	968.6 ± 3.1	971.3 ± 5.4*	21.0 ± 2.5	17.1 ± 3.6*	86.4 ± 8.7	84.3 ± 8.7
Tokorozawa city	4.3 ± 1.6	6.6 ± 3.8*	6.5 ± 3.3	7.9 ± 3.3	30.7 ± 16.2	36.1 ± 16.0	1006.0 ± 3.3	1009.1 ± 5.8*	22.6 ± 2.7	19.0 ± 3.7*	78.6 ± 11.0	76.2 ± 10.4
Hanno city	4.6 ± 1.7	6.7 ± 3.9*	7.1 ± 3.7	8.8 ± 3.7	29.4 ± 15.1	35.4 ± 16.1	998.6 ± 3.3	1001.6 ± 5.8*	22.5 ± 2.7	18.8 ± 3.8*	80.1 ± 10.8	77.0 ± 9.9
Fukaya city	4.2 ± 1.6	6.6 ± 3.7*	6.6 ± 3.5	7.9 ± 3.9	31.7 ± 15.3	36.8 ± 16.5	1008.3 ± 3.3	1011.6 ± 5.7*	22.8 ± 2.7	19.0 ± 3.7*	79.7 ± 10.0	77.5 ± 9.8
Katsuura city	4.2 ± 1.7	6.5 ± 4.6*	5.4 ± 1.6	6.0 ± 2.2	24.2 ± 10.9	26.9 ± 11.1	1007.9 ± 3.1	1010.9 ± 6.3*	23.5 ± 2.1	20.0 ± 3.5*	84.1 ± 6.8	80.4 ± 9.5
Kujukuri town	3.9 ± 1.5	6.4 ± 4.4*	6.6 ± 2.6	7.7 ± 3.1	25.7 ± 11.3	28.4 ± 11.1	1013.1 ± 3.2	1016.2 ± 6.2*	22.7 ± 2.3	19.0 ± 3.4*	83.1 ± 7.5	78.8 ± 9.1
Sammu city	3.9 ± 1.6	6.4 ± 4.4*	6.8 ± 2.7	7.7 ± 3.0	28.5 ± 13.7	32.8 ± 12.2	1011.6 ± 3.2	1014.7 ± 6.1*	22.7 ± 2.3	19.0 ± 3.4*	85.5 ± 7.6	81.6 ± 8.6
Tateyama city	3.8 ± 1.5	6.3 ± 4.1*	6.2 ± 2.1	6.9 ± 2.7	29.8 ± 12.4	32.9 ± 10.7	1009.5 ± 3.1	1012.6 ± 6.3*	23.8 ± 2.2	20.2 ± 3.3*	81.2 ± 7.1	77.6 ± 9.0
Chuo ward, Chiba city	3.9 ± 1.5	6.5 ± 4.0*	5.9 ± 2.0	6.7 ± 2.4	27.4 ± 12.2	31.1 ± 11.9	1012.8 ± 3.2	1016.1 ± 6.1*	23.4 ± 2.5	20.0 ± 3.5*	75.0 ± 9.2	70.0 ± 9.9
Choshi city	3.8 ± 1.5	6.5 ± 4.6*	3.9 ± 1.6	4.6 ± 2.1	22.8 ± 9.4	27.4 ± 11.0	1012.4 ± 3.2	1015.4 ± 6.3*	23.7 ± 1.7	20.7 ± 3.1*	84.5 ± 7.8	79.1 ± 9.9*
Futtsu city	3.8 ± 1.5	6.5 ± 3.8*	6.5 ± 2.7	7.2 ± 2.9	23.8 ± 11.6	27.5 ± 10.7	1010.6 ± 3.2	1013.8 ± 6.1*	23.3 ± 2.5	19.8 ± 3.4*	82.4 ± 8.3	77.8 ± 9.1
Narita city	3.8 ± 1.6	6.5 ± 4.1*	6.1 ± 2.4	7.4 ± 2.9	29.0 ± 13.3	34.0 ± 12.4	1011.4 ± 3.2	1014.6 ± 6.0*	22.4 ± 2.4	18.8 ± 3.4*	84.1 ± 8.1	79.6 ± 8.5*
Mobara city	4.0 ± 1.6	6.4 ± 4.3*	6.6 ± 2.5	7.8 ± 3.2	30.3 ± 14.4	35.3 ± 13.6	1010.2 ± 3.1	1013.3 ± 6.2*	22.9 ± 2.3	19.2 ± 3.4*	83.1 ± 8.3	79.3 ± 9.8

Values are presented as mean ± SD. *n* = 28. *p < 0.05(unpaired *t*-test)

### Effect of kaempferol on symptom frequency

**Fig. 3:**
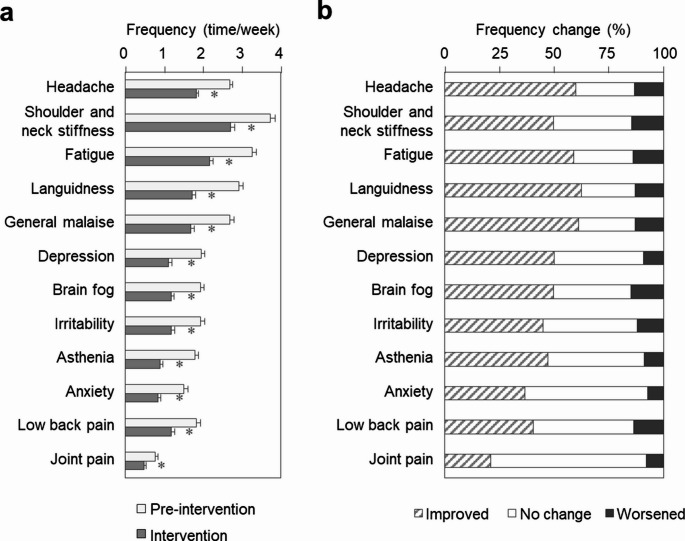
Frequency of subjective weather-related discomfort symptoms. (**a**) Weekly frequency of each subjective symptom—including headache, shoulder and neck stiffness, fatigue, languidness, general malaise, depression, brain fog, irritability, asthenia, anxiety, low back pain, and joint pain—during the pre-intervention and intervention periods. Values are presented as mean ± SE; *n* = 387. *p < 0.05 (paired *t*-test). (**b**) Percentage of participants who reported a reduction in the weekly frequency of each symptom between the pre-intervention and intervention periods. *n* = 387

The frequency of all assessed subjective symptoms—including headache, shoulder and neck stiffness, fatigue, languidness, general malaise, depression, brain fog, irritability, asthenia, anxiety, low back pain, and joint pain—was significantly reduced during the intervention period compared to the pre-intervention period (Fig. 3a, Supplementary Table 1). The proportion of participants with changes in symptom frequency is shown in Fig. 3b. Notably, 60% of participants reported reduced headache frequency, followed by 63%, 61%, and 59% for languidness, general malaise, and fatigue, respectively.

### Effect of kaempferol on symptom duration

**Fig. 4:**
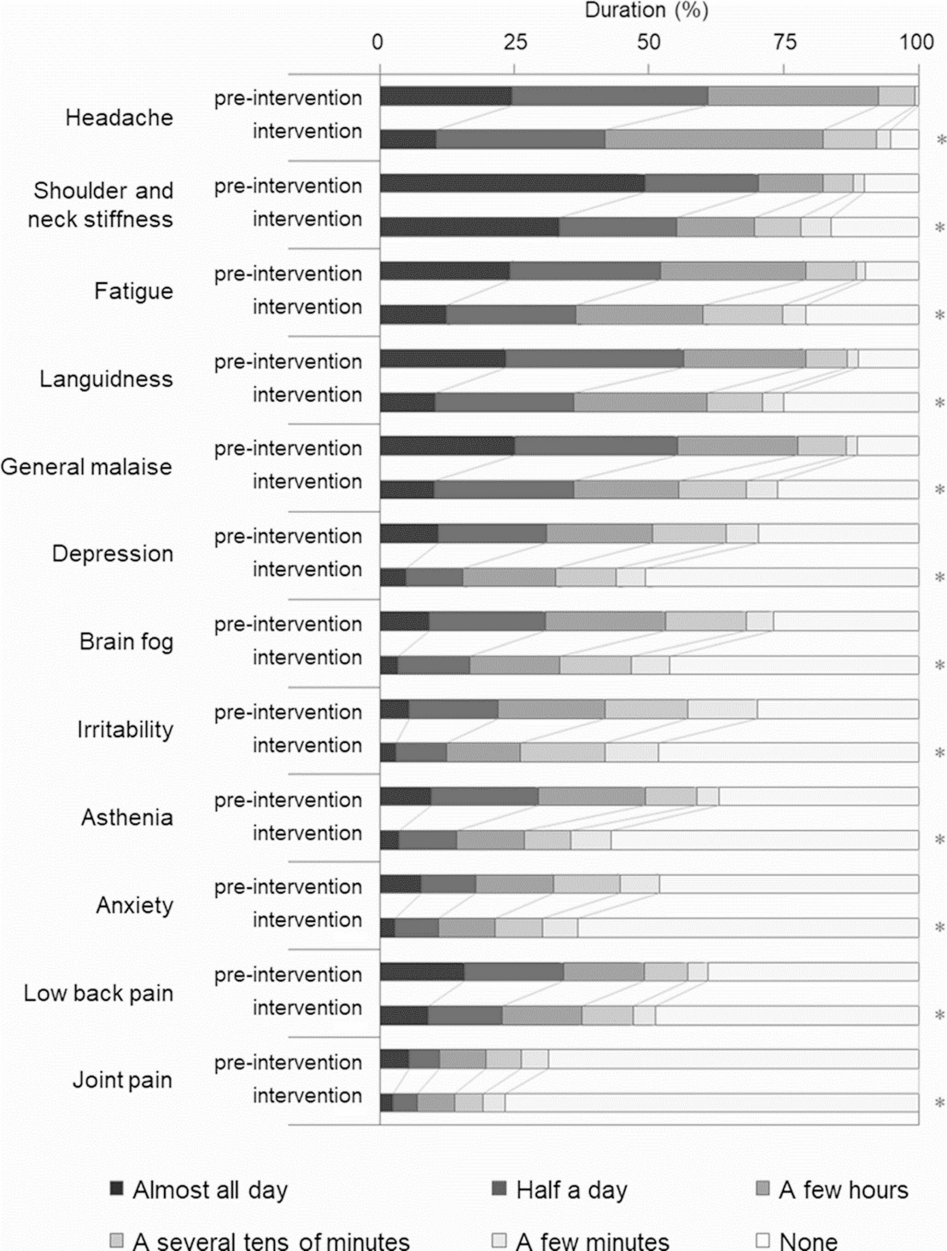
Duration of subjective weather-related discomfort symptoms. Daily duration of each symptom—including headache, shoulder and neck stiffness, fatigue, languidness, general malaise, depression, brain fog, irritability, asthenia, anxiety, low back pain, and joint pain—during the pre-intervention and intervention periods. *n* = 387. *p < 0.05 (Wilcoxon signed-rank test)

Symptom duration also significantly decreased during the intervention period for all symptoms (Fig. 4, Supplementary Table 2). The number of participants reporting symptoms lasting “almost all day” or “half a day” significantly declined for all symptoms except joint pain (Supplementary Table 3). Conversely, the proportion of participants reporting “none” for each symptom significantly increased during the intervention period (Supplementary Table 4).

### Effect of kaempferol on symptom severity

**Fig. 5:**
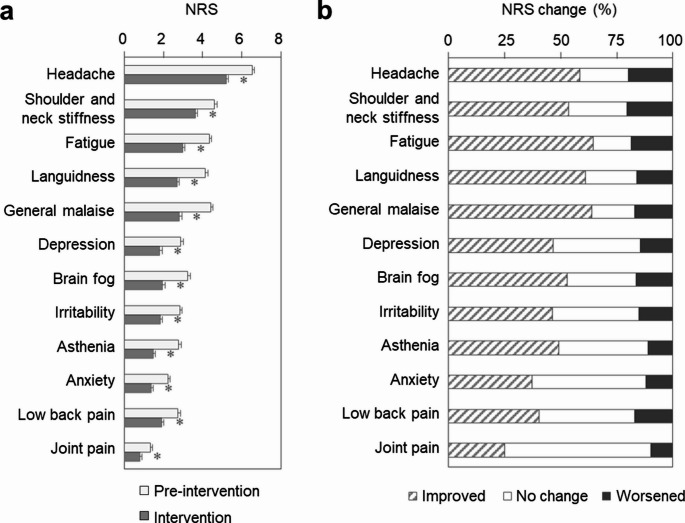
Severity of subjective weather-related discomfort symptoms.(**a**) Severity of each symptom—including headache, shoulder and neck stiffness, fatigue, languidness, general malaise, depression, brain fog, irritability, asthenia, anxiety, low back pain, and joint pain—measured using the NRS during the pre-intervention and intervention periods. Values are presented as mean ± SE; *n* = 387. *p < 0.05 (paired *t*-test). (**b**) Percentage of participants who reported a reduction in NRS scores for each symptom between the pre-intervention and intervention periods. *n* = 387

Severity scores for all symptoms, as measured using the NRS, were significantly lower during the intervention period than during the pre-intervention period (Fig. 5a, Supplementary Table 5). Among participants, 59% reported a reduction in headache severity, with similar improvements observed for fatigue (65%), general malaise (64%), and languidness (61%) (Fig. 5b). Additionally, subgroup analysis showed that improvements in headache—the most frequently reported symptom of weather-related pain—were statistically significant across all parameters (frequency, duration, and severity) in both male and female participants (Supplementary Table 6). Furthermore, female participants showed significant improvements across all other symptoms and measures, whereas improvements in the male group were more limited. Improvements across all other symptoms were more pronounced in female participants.

### Overall symptom improvement

**Fig. 6:**
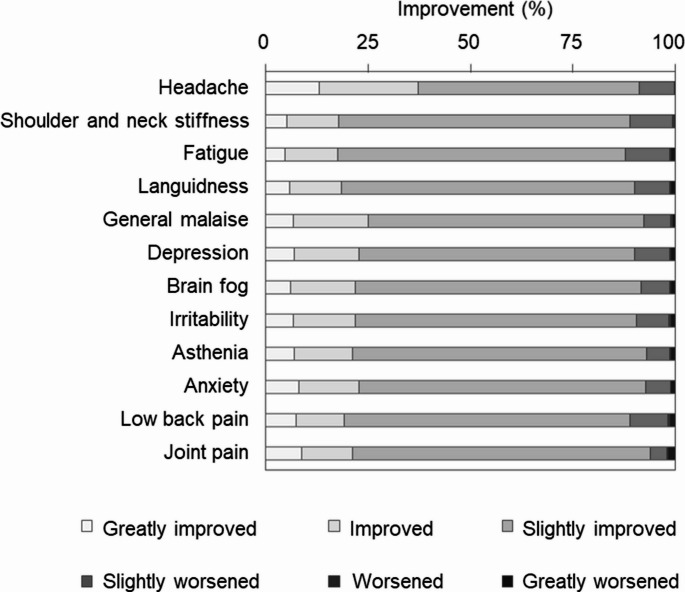
Degree of improvement in subjective weather-related discomfort symptoms. Self-reported degree of improvement in each symptom—including headache, shoulders and neck stiffness, fatigue, languidness, general malaise, depression, brain fog, irritability, asthenia, anxiety, low back pain, and joint pain—between the pre-intervention and intervention periods. *n* = 387

At the end of the intervention, over 80% of participants reported some degree of improvement in their overall symptoms, categorized as “greatly improved,” “improved,” or “slightly improved” (Fig. 6).

## Discussion

Although weather-related pain affects many individuals worldwide, significantly impairing daily life and posing a major societal concern (Sato et al. 2021), weather remains an uncontrollable environmental factor. Thus, mitigating the physiological responses triggered by weather fluctuations is essential for alleviating such symptoms. However, the underlying mechanisms remain poorly understood. We hypothesized that a slight decrease in oxygen supply to peripheral tissues due to low barometric pressure may contribute to the onset of weather-related pain and that targeting this physiological mechanism could offer a novel therapeutic approach. Accordingly, this study examined whether kaempferol—a flavonoid known to enhance oxygen utilization efficiency and regulate autonomic nervous system balance—may help reduce subjective symptoms associated with weather-related discomfort. Notably, despite greater barometric pressure fluctuations during the intervention period than during the pre-intervention period, participants reported significant reductions in the frequency, duration, and severity of subjective symptoms. These results support the potential effectiveness of daily kaempferol intake in managing weather-related pain. Furthermore, consistent with our previous studies (Akiyama et al. 2023; Ikeda et al. 2024a, b; Okita et al. 2024, 2025), no adverse effects were observed with kaempferol intake in the present study.

This study revealed that kaempferol intake may improve various subjective physical and mental discomfort symptoms. Notably, headache, fatigue, languidness, and general malaise—symptoms reported by more than 85% of participants in the background data—showed consistent reductions in frequency, duration, and severity. While weather-related pain symptoms vary among individuals, these common symptoms provide a useful basis for exploring the mechanisms by which kaempferol may exert its effects. For instance, headache is not only a hallmark of weather-related pain but also commonly observed in high-altitude environments, where its severity is influenced by changes in barometric pressure. Headache is also closely associated with hypoxia and oxygen utilization (Georges et al. 2022). Supporting this, Hagen et al. (2015) reported that individuals who experience headaches exhibit lower maximal oxygen uptake than those without headaches. Moreover, autonomic nervous system dysfunction has been implicated in headache pathophysiology (Gevirtz 2022). Fatigue, another prevalent symptom, is frequently linked to increased physical and mental burden and is reflected in elevated low frequency to high frequency (LF/HF) ratio, indicating autonomic nervous system imbalance (Tanaka et al. 2011). Similarly, languidness has been associated with reduced blood flow, lower oxygen availability, and inhibited metabolism activity (Ishihara 2005; Ishihara et al. 2014).

Previous studies have shown that kaempferol intake enhances maximal oxygen uptake and oxygen utilization efficiency (Ikeda et al. 2024b), reduces cardiopulmonary load, alleviates muscle damage, and lowers perceived exertion, thereby alleviating both physical and mental fatigue (Ikeda et al. 2024a; Okita et al. 2024). Kaempferol also reduces the resting LF/HF ratio (Ikeda et al. 2024a) and enhances metabolism (Akiyama et al. 2022). These physiological effects may have worked synergistically to improve the subjective weather-related pain observed in this study. Furthermore, the symptoms may be interconnected and collectively contribute to overall discomfort, including general malaise. Regarding mental symptoms, the fear–avoidance model posits that negative thoughts about pain can intensify fear, leading to unnecessary rest, depression, activity restriction, and further pain amplification (Vlaeyen and Linton 2000). It is plausible that symptom relief from kaempferol disrupted this cycle, indirectly improving mental well-being. However, mental symptoms are also influenced by the accumulation of past pain experiences, which may weaken the intervention’s impact in some individuals. Additionally, since the fear-avoidance model is primarily relevant to chronic pain, its applicability to transient weather-related pain may be limited. Joint pain, which was the least commonly reported symptom at baseline, may have shown smaller improvements for this reason. While definitive conclusions cannot be drawn, these findings suggest that kaempferol intake alleviates subjective weather-related discomfort symptoms, either directly or indirectly, primarily by enhancing oxygen utilization efficiency. If peripheral hypoxia is the fundamental cause, it represents a common biological mechanism shared by all individuals from various backgrounds. Therefore, kaempferol intake could be effective for all symptoms arising from hypoxia, not only weather-related pain. However, since females are more sensitive to weather-related pain than males, although this remains within the realm of personal opinion, they may be more prone to experiencing peripheral hypoxia. Therefore, kaempferol may have led to more effective symptom improvement in females than in males.

In a previous study, the migraine preventive drug eptinezumab reduced headache severity by 3.0 points on the NRS score after 3 weeks of treatment in patients with chronic headaches (Barbanti et al. 2024). In comparison, the present study observed a 1.2-point reduction in NRS scores following kaempferol intake. According to established criteria, a 1.0–2.0-point decrease is considered “slightly improved,” whereas a reduction greater than 2.0 points is classified as “much improved” (Salaffi et al. 2004). These results suggest that kaempferol offers a foundational dietary strategy for managing weather-related pain, with the added benefit of being a safe, non-pharmacological option. Additionally, because kaempferol and pharmacological drugs, including eptinezumab, operate via different mechanisms, they may be used in a complementary manner to enhance therapeutic outcomes.

While this study suggests that kaempferol helps alleviate various subjective discomforts associated with weather-related pain, several limitations should be acknowledged. First, the open-label, pre–post comparison design without a placebo control introduces potential biases, including placebo effects, regression to the mean, the Hawthorne effect, and seasonal variations. To address these confounding factors, future studies should adopt a randomized, double-blind, placebo-controlled crossover design. Second, because this study was conducted under natural weather conditions, controlling for environmental variability across participants was difficult. Future research conducted under artificially controlled weather conditions—or using wearable devices and smartphone applications to continuously monitor symptoms and weather exposure—could provide more robust and objective data. Third, the reliance on self-reported questionnaires introduces inherent subjective bias. Moreover, the proposed mechanisms remain hypothetical. As no objective biomarkers for weather-related pain currently exist, future studies should incorporate physiological indicators, including autonomic nervous system activity, vertigo thresholds via inner ear stimulation, LF/HF ratio, and heart rate. Clinical assessments by medical professionals could also enhance the reliability of symptom evaluation. Fourth, the study population comprised exclusively Japanese individuals, with approximately 90% identifying as female. Although symptom improvement was observed across the sexes, the generalizability of the findings is limited. Integrating these approaches with physiological measurements and sex-stratified analyses in more diverse populations would enable a deeper understanding of potential sex-based differences in susceptibility to weather-related pain. Despite these limitations, a key strength of this study lies in its exploration of a safe, food-based intervention for a condition that currently lacks fundamental treatment options. With further validation, daily kaempferol intake may offer a practical and accessible strategy to improve quality of life and support public health.

## Conclusions

This study suggests that daily intake of kaempferol helps reduce the frequency, duration, and severity of various subjective physical and mental symptoms associated with weather-related pain. As a safe and easily consumable food-derived compound, kaempferol holds promise as a practical dietary supplement for individuals seeking to manage such symptoms. Future investigations should aim to clarify the underlying mechanisms of action by incorporating objective physiological assessments and conducting studies under controlled experimental conditions. Establishing an effective and accessible strategy for managing weather-related pain could contribute to an improved quality of life on a global scale—reducing absenteeism, enhancing productivity, and supporting overall well-being.

## Electronic supplementary material

Below is the link to the electronic supplementary material.

Supplementary Material 1.

## Data Availability

The data that support the findings of this study are available from the corresponding author upon reasonable request.

## Abbreviations

mHBP: micro-high barometric pressure
NRS: numerical rating scale
CI: confidence interval
SD: standard deviation
SE: standard error
LF/HF: low frequency to high frequency
