# Supplementary material for: Effects of kaempferol on weather-related pain: an open-label pilot study of subjective headache and other discomforts in pre-intervention and intervention periods in Japan
Source: Int J Biometeorol. 2025 Jul 24;69(10):2697–709. doi: 10.1007/s00484-025-02985-6 (PMC12540546; doi:10.1007/s00484-025-02985-6)
Supplement: Supplementary file 1 — Supplementary Material 1. [file 484_2025_2985_MOESM1_ESM.docx]

**Title**

Effects of Kaempferol on Weather-related Pain: An Open-label Pilot Study of Subjective Headache and Other Discomforts in Pre-intervention and Intervention Periods in Japan

Yasutaka Ikeda^1^, Moe Yamamoto^1^, Aina Gotoh-Katoh^1^, Shoichiro Inoue^1^, and Jun Sato^2^

^1^Advanced Research Institute for Core Science, Otsuka Pharmaceutical Co., Ltd., 2-16-4, Konan, Minato-ku, Tokyo 108-8242, Japan

^2^Department of Pain Medicine, Aichi Medical University, 1-1 Yazako-Karimata, Nagakute, Aichi 480-1195, Japan

**Corresponding author**

Jun Sato

Department of Pain Medicine, Aichi Medical University, 1-1 Yazako-Karimata, Nagakute, Aichi 480-1195, Japan

[jsato0310@aichi-med-u.ac.jp](mailto:jsato0310@aichi-med-u.ac.jp)

**Supplementary Table 1** Statistical analysis data of frequency of subjective weather-related discomfort symptoms (paired *t*-test).

| Symptom | Cohen’s *d* (95% CI) | *p*_corr_ |
| --- | --- | --- |
| Headache | 0.61 (0.50–0.72) | <0.001 |
| Shoulder and neck stiffness | 0.50 (0.39–0.60) | <0.001 |
| Fatigue | 0.63 (0.52–0.74) | <0.001 |
| Languidness | 0.67 (0.56–0.78) | <0.001 |
| General malaise | 0.62 (0.51–0.72) | <0.001 |
| Depression | 0.54 (0.44–0.65) | <0.001 |
| Brain fog | 0.47 (0.37–0.58) | <0.001 |
| Irritability | 0.51 (0.40–0.61) | <0.001 |
| Asthenia | 0.56 (0.45–0.67) | <0.001 |
| Anxiety | 0.43 (0.32–0.53) | <0.001 |
| Low back pain | 0.38 (0.27–0.48) | <0.001 |
| Joint pain | 0.22 (0.12–0.32) | <0.001 |

CI, confidence interval; *p*_corr_, *p*-value adjusted using the Bonferroni correction.

**Supplementary Table 2** Statistical analysis data of duration of subjective weather-related discomfort symptoms (Wilcoxon signed-rank test).

| Symptom | rank-biserial correlation (95% CI) | *p*_corr_ |
| --- | --- | --- |
| Headache | 0.64 (0.57–0.70) | <0.001 |
| Shoulder and neck stiffness | 0.57 (0.48–0.64) | <0.001 |
| Fatigue | 0.59 (0.51–0.67) | <0.001 |
| Languidness | 0.63 (0.55–0.70) | <0.001 |
| General malaise | 0.67 (0.59–0.73) | <0.001 |
| Depression | 0.70 (0.63–0.77) | <0.001 |
| Brain fog | 0.64 (0.55–0.71) | <0.001 |
| Irritability | 0.58 (0.48–0.66) | <0.001 |
| Asthenia | 0.76 (0.69–0.81) | <0.001 |
| Anxiety | 0.62 (0.51–0.71) | <0.001 |
| Low back pain | 0.43 (0.31–0.54) | <0.001 |
| Joint pain | 0.39 (0.20–0.55) | 0.003 |

CI, confidence interval; *p*_corr_, *p*-value adjusted using the Bonferroni correction.

**Supplementary Table 3** Statistical analysis data for reduction in duration of subjective weather-related discomfort symptoms; “almost all day” and “half a day” (McNemar’s test).

| Symptom | Decrease (%) | OR (95% CI) | *p*_corr_ |
| --- | --- | --- | --- |
| Headache | 19 | 3.47 (2.29–5.39) | <0.001 |
| Shoulder and neck stiffness | 15 | 3.68 (2.28–6.20) | <0.001 |
| Fatigue | 16 | 3.03 (1.99–4.75) | <0.001 |
| Languidness | 20 | 4.04 (2.61–6.47) | <0.001 |
| General malaise | 19 | 4.00 (2.56–6.47) | <0.001 |
| Depression | 16 | 5.62 (3.09–11.05) | <0.001 |
| Brain fog | 14 | 4.00 (2.36–7.13) | <0.001 |
| Irritability | 10 | 3.18 (1.81–5.84) | <0.001 |
| Asthenia | 15 | 6.90 (3.54–15.02) | <0.001 |
| Anxiety | 7 | 4.00 (1.89–9.44) | <0.001 |
| Low back pain | 11 | 2.91 (1.79–4.90) | <0.001 |
| Joint pain | 4 | 2.07 (1.08–4.12) | 0.311 |

OR, Odds ratios; CI, confidence interval; *p*_corr_, *p*-value adjusted using the Bonferroni correction.

**Supplementary Table 4** Statistical analysis data for an increase in the duration of subjective weather-related discomfort symptoms;” none” (McNemar’s test).

| Symptom | Increase (%) | OR (95% CI) | *p*_corr_ |
| --- | --- | --- | --- |
| Headache | 5 | 0.00 (0.00–0.20) | <0.001 |
| Shoulder and neck stiffness | 6 | 0.35 (0.17–0.68) | 0.011 |
| Fatigue | 11 | 0.22 (0.11–0.41) | <0.001 |
| Languidness | 14 | 0.22 (0.12–0.38) | <0.001 |
| General malaise | 15 | 0.15 (0.07–0.29) | <0.001 |
| Depression | 21 | 0.12 (0.06–0.22) | <0.001 |
| Brain fog | 19 | 0.23 (0.14–0.36) | <0.001 |
| Irritability | 18 | 0.14 (0.07–0.27) | <0.001 |
| Asthenia | 20 | 0.13 (0.07–0.24) | <0.001 |
| Anxiety | 15 | 0.18 (0.09–0.33) | <0.001 |
| Low back pain | 10 | 0.43 (0.27–0.68) | 0.002 |
| Joint pain | 8 | 0.43 (0.25–0.71) | 0.006 |

OR, Odds ratios; CI, confidence interval; *p*_corr_, *p*-value adjusted using the Bonferroni correction.

**Supplementary Table 5** Statistical analysis data of severity of subjective weather-related discomfort symptoms (paired *t*-test).

| Symptom | Cohen’s *d* (95% CI) | *p*_corr_ |
| --- | --- | --- |
| Headache | 0.57 (0.46–0.67) | <0.001 |
| Shoulder and neck stiffness | 0.41 (0.31–0.52) | <0.001 |
| Fatigue | 0.58 (0.47–0.68) | <0.001 |
| Languidness | 0.63 (0.52–0.74) | <0.001 |
| General malaise | 0.61 (0.50–0.72) | <0.001 |
| Depression | 0.49 (0.39–0.60) | <0.001 |
| Brain fog | 0.51 (0.40–0.61) | <0.001 |
| Irritability | 0.45 (0.35–0.56) | <0.001 |
| Asthenia | 0.57 (0.46–0.67) | <0.001 |
| Anxiety | 0.40 (0.30–0.50) | <0.001 |
| Low back pain | 0.37 (0.26–0.47) | <0.001 |
| Joint pain | 0.27 (0.17–0.37) | <0.001 |

CI, confidence interval; *p*_corr_, *p*-value adjusted using the Bonferroni correction.

**Supplementary Table 6** Corrected *p*-values for the frequency, duration, and severity of each symptom in female and male groups (frequency and severity, paired *t*-test; duration, Wilcoxon signed-rank test).

| Symptom | Frequency | | Duration | | Severity | |
| --- | --- | --- | --- | --- | --- | --- |
|  | Female | Male | Female | Male | Female | Male |
| Headache | <0.001 | 0.003 | <0.001 | 0.002 | <0.001 | <0.001 |
| Shoulder and neck stiffness | <0.001 | 0.061 | <0.001 | 1.000 | <0.001 | 0.934 |
| Fatigue | <0.001 | 0.011 | <0.001 | 0.008 | <0.001 | 0.041 |
| Languidness | <0.001 | 0.006 | <0.001 | 0.318 | <0.001 | 0.864 |
| General malaise | <0.001 | 0.301 | <0.001 | 0.051 | <0.001 | 0.041 |
| Depression | <0.001 | 0.199 | <0.001 | 0.040 | <0.001 | 0.137 |
| Brain fog | <0.001 | 0.105 | <0.001 | 0.133 | <0.001 | 0.003 |
| Irritability | <0.001 | 0.178 | <0.001 | 1.000 | <0.001 | 1.000 |
| Asthenia | <0.001 | 0.010 | <0.001 | 0.011 | <0.001 | <0.001 |
| Anxiety | <0.001 | 0.030 | <0.001 | 1.000 | <0.001 | 0.124 |
| Low back pain | <0.001 | 0.005 | <0.001 | 0.028 | <0.001 | 0.001 |
| Joint pain | <0.001 | 1.000 | <0.001 | 1.000 | <0.001 | 1.000 |
